# Complete mitochondrial genome of the *Acentrogobius pflaumii* (Gobiiformes, Gobiidae) with phylogenetic consideration

**DOI:** 10.1080/23802359.2020.1715283

**Published:** 2020-01-29

**Authors:** Hao Wu, Meiya Yu, Lei Zhou

**Affiliations:** Institute of Biopharmaceutical, Zhejiang Pharmaceutical College, Ningbo, China

**Keywords:** *Acentrogobius pflaumii*., mitochondrion, genome

## Abstract

The complete mitochondrial genome sequence of *Acentrogobius pflaumii* was sequenced. It was 16,515-nucleotide in length and consisted of 13 protein-coding genes, 2 ribosomal RNA genes, 22 transfer RNA genes, and two non-coding regions (L-strand replication origin and control region), showing conserved gene arrangement with most vertebrates. The phylogenetic analysis based on complete mitochondrial genome of *A. pflaumii* as well as other Gobiiformes species showed that *A. pflaumii* had close relationships with *Amoya chusanensis*. This study will provide useful genetic informatin for future phylogenetic and taxonomic classification of gobiiformes.

*Acentrogobius pflaumii* is the largest amphibious teleost fishes and they are more and more attracting attentions of environmental experts and the public. In fact, it is a common fish in the coastal waters from east of china sea (Yi et al. 2016). There is no report of the complete genome of this fish *A. pflaumii*, which was developed in Zhoushan, Zhejiang Province, China (N29°94′95″, E122°18′03″) in October 2019. Therefore, it is very important to characterize the complete mitogenome of this species, which can be utilized in research on taxonomic resolution, population genetic structure and phylogeography, and phylogenetic relationship. Total DNA was extracted from muscle following TIANamp Marine Animals DNA Kit (Tiangen, China), and NOVOPlasty software was used to assemble the mitogenomes, the mistake parameter was set by default (Dierckxsens et al. 2017). The samples were stored in −80 °C in Key Lab of Institute of Biopharmaceutical, Zhejiang Pharmaceutical College Ningbo, China. Number is AP-1.

Finally, a physical map of *A. pflaumii* mitogenome was generated and uploaded to GenBank with accession number MN745094.The complete mitogenome of *A. pflaumii* was 16,515 bp in length. The genomic organization was identical to those of typical vertebrate mitochondrial genomes, including two rRNA genes, 13 protein-coding genes, 22 tRNA genes, a light-strand replication origin (OL), and a putative control region (CR). The overall base composition was 26.6% of A, 25.8% of T, 29.3% of C, and 18.3% of G with a slight A + T bias (52.4%) like other vertebrate mitochondrial genomes.

Most of the genes were encoded on the heavy strand (H-strand), except for eight tRNA genes (tRNA-Gln, tRNA-Ala, tRNA-Asn, tRNA-Cys, tRNA-Tyr, tRNA-Ser, tRNA-Glu, and tRNA-Pro), origin region as well as ND6, which were encoded on the L-strand. For the 13 protein-coding genes, 11 genes started with ATG while only *COI* started with GTG and *ND4L* started with TTA. The two ribosomal RNA genes, 12SrRNA (946 bp) and 16SrRNA (1,672 bp), were located between tRNA-Phe and tRNA-Leu, separated by tRNA-Val gene. As in other vertebrates, *A. pflaumii* had two non-coding regions, the L-strand replication origin region (36 bp) located between tRNA-Asn and tRNA-Cys, and the control region located within the tRNA-Pro and tRNA-Phe.

The phylogenetic tree showed that the *A. pflaumii* has the closer relationship with *Amoya chusanensis* (Figure 1). The phylogeny was reconstructed based on the General Time Reversible + Invariant + gamma sites (GTR + I + G) model of nucleotide substitution using Mega7 (Kumar et al. 2016). The complete mitochondrial genome sequence of the *A. pflaumii* provided an important dataset for a better understanding of the mitogenomic diversities and evolution in fish as well as novel genetic markers for studying population genetics and species identification.

**Figure 1.**
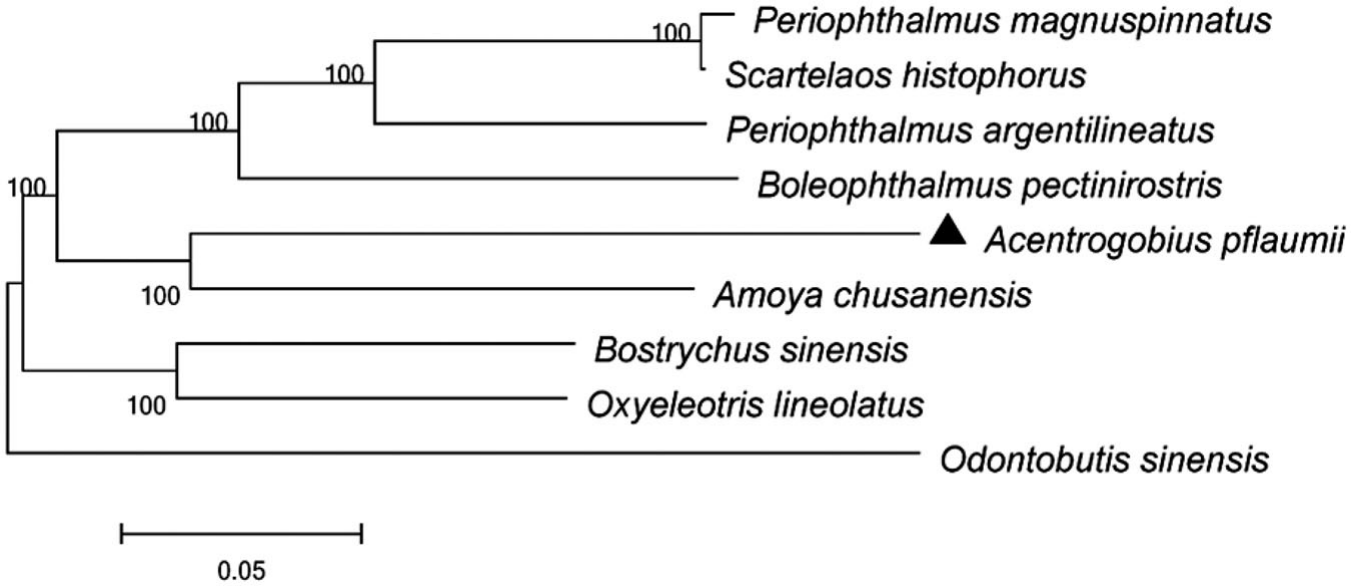
The phylogenetic relationship was estimated using the Maximum Likelihood method for the complete mitochondrial genome. Genbank accession Numbers: *Amoya chusanensis* (KC196075), *Boleophthalmus pectinirostri*s (NC_016195), *Bostrychus sinensis* (AP019357), *Odontobutis sinensis* (NC_022818), *Oxyeleotris lineolatus* (KP663727), *Periophthalmus argentilineatus* (NC_029368), *Periophthalmus magnuspinnatus* (KT 357639), *Scartelaos histophorus* (JQ654459), *Acentrogobius pflaumii* (MN745094). The numbers at the nodes are bootstrap percent probability values based on 1000 replications.
